# Analysis of *Escherichia coli *nicotinate mononucleotide adenylyltransferase mutants *in vivo *and *in vitro*

**DOI:** 10.1186/1471-2091-6-16

**Published:** 2005-09-09

**Authors:** Martin Stancek, Robert Schnell, Monica Rydén-Aulin

**Affiliations:** 1 Department of Genetics, Microbiology and Toxicology, Stockholm University, S-106 91 Stockholm, Sweden; 2 In vitro Sweden AB, Box 21160, S-100 31 Stockholm, Sweden; 3 Department of Medical Biochemistry and Biophysics, Karolinska Institutet, S-171 77 Stockholm, Sweden

## Abstract

**Background:**

Adenylation of nicotinate mononucleotide to nicotinate adenine dinucleotide is the penultimate step in NAD^+ ^synthesis. In *Escherichia coli*, the enzyme nicotinate mononucleotide adenylyltransferase is encoded by the *nadD *gene. We have earlier made an initial characterization *in vivo *of two mutant enzymes, NadD72 and NadD74. Strains with either mutation have decreased intracellular levels of NAD^+^, especially for one of the alleles, *nadD72*.

**Results:**

In this study these two mutant proteins have been further characterized together with ten new mutant variants. Of the, in total, twelve mutations four are in a conserved motif in the C-terminus and eight are in the active site. We have tested the activity of the enzymes *in vitro *and their effect on the growth phenotype *in vivo*. There is a very good correlation between the two data sets.

**Conclusion:**

The mutations in the C-terminus did not reveal any function for the conserved motif. On the other hand, our data has lead us to assign amino acid residues His-19, Arg-46 and Asp-109 to the active site. We have also shown that the *nadD *gene is essential for growth in *E. coli*.

## Background

Biosynthesis of nicotinamide adenine dinucleotides plays a central role in the metabolism of all organisms [1,2]. Their primary function is to serve as either donors or acceptors in biochemical oxidation-reduction reactions. The nucleotides can also be used as substrates in non-redox reactions *e.g*. ADP ribosylation [3], biosynthesis of cyclic ADP-ribose [4], and as a dehydrating agent for DNA ligase [5].

**Figure 1:**
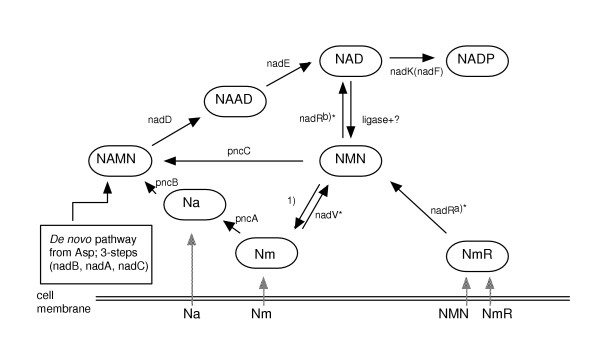
Biosynthesis of NAD in bacteria. Abbreviations: Na, nicotinic acid; Nm, nicotinamide; NMN, nicotinamide mononucleotide; NAMN, nicotinic acid mononucleotide; NmR, nicotinamide ribonucleoside; NAAD, nicotinic acid adenine dinucleotide; NAD, nicotinamide adenine dinucleotide; NADP, nicotinamide adenine dinucleotide phosphate. Enzymes: *nadA *codes for quinolinate synthase; *nadB *L-aspartate oxidase; *nadC *quinolinic acid phosphoribosyltransferase; *nadD *nicotinic acid mononucleotide adenylyltransferase; *nadE *NAD synthetase; *nadK *(*nadF*) NAD kinase; *pncA *nicotinamide deamidase; *pncB *nicotinic acid phosphoribosyltransferase; *pncC *NMN deamidase; nadV* Nm phosphoribosyltransferase (in *Haemophilus ducreyi*); *ligase+? *DNA ligase, only partially responsible for the activity; *nadR *bifunctional activity; a)* N-ribosylnicotinamide kinase, b)* NMN adenylyltransferase (in *H. influenzae*); 1) NMN glycohydrolase (gene not yet identified). Some of these steps may occur in the periplasmic space or at the inner membrane. Asterisks indicate activities not identified *in vivo *in *E. coli*.

There are several metabolic pathways for biosynthesis of nicotinamide adenine dinucleotide (NAD^+^) in bacteria (Figure 1). The *de novo *pathway consists of five steps; it starts with the oxidation of aspartate to iminosuccinic acid, which in turn reacts with dihydroxyacetone phosphate to give quinolinic acid (QA). QA is phosphoribosylated and decarboxylated resulting in nicotinic acid mononucleotide (NAMN). NAMN is adenylated to nicotinic acid adenine dinucleotide (NAAD), which in turn is amidated to complete NAD^+ ^biosynthesis. The genes coding for the different enzymes in *Escherichia coli *(*E. coli*) have been identified [6,7].

Besides *de novo *synthesis of NAD^+ ^there are several salvage pathways where exogenous precursors are converted to NAMN, which then can be converted to NAD^+^. This means that all the steps leading to NAMN are nonessential. On the contrary, in the last two reactions, NAMN to NAAD and NAAD to NAD^+^, metabolites cannot be supplied from outside the cell. Thus, the enzymes nicotinic acid mononucleotide adenylyltransferase (NAMNAT) and NAD synthetase are essential for growth of *E. coli *(coded for by the genes *nadD *and *nadE*, respectively). However, if the *nadD *gene is essential for growth has been questioned. In a survey with transposon insertions into metabolic genes, insertions into the *nadD *gene were found, at the same time the authors show that the entire gene cannot be deleted [8]. It should be pointed out that the insertion site in the *nadD *gene was not shown.

The *E. coli nadD *gene has been identified [9] and the crystal structure of the enzyme has been solved [10]. The enzyme has a molecular weight of 24.5 kD and unlike the counterparts in Archaea and Eukarya it is suggested to function as a monomer. The *E. coli *enzyme shows strong substrate preference for NAMN rather than for nicotinamide mononucleotide (NMN), this is in contrast to the human and archaeal enzymes [9]. NAMNAT is a member of the nucleotidyltransferase super family [11], that includes ATP surfurylase, cytidyltransferase, pantothenate synthetase, and class I tRNA synthetases. The group is characterized by a modified dinucleotide-binding fold (Rossmann-fold) and by the presence of a conserved ATP binding motif, T/HXGH [11]. The importance of the latter has been shown [12-14].

We have earlier shown that two mutations in the *nadD *gene (*nadD72 *and *nadD74*) lead to decreased levels of NAD^+ ^in the cell [15]. This, in turn, leads to temperature sensitive growth on synthetic minimal medium for both mutants. A strain with the *nadD72 *mutation is most severely affected and has additional phenotypic changes like; complete inability to grow on minimal medium and temperature sensitive growth on rich medium. Thus, a link between NAD^+ ^synthesis and ability to grow on different substrates at different temperatures was found.

In this paper we have shown that the *nadD *gene is essential for growth and we have extended the analysis of the enzyme by creating ten additional mutants. Seven of them have mutated residues close to or in the active site, as is *nadD74*, and three were created to study the role of the C-terminus, which is affected by *nadD72*. All twelve mutant proteins were analyzed *in vitro *for activity and *in vivo *for their effect on the growth phenotype. The two data sets correlate very well. We have not been able to find a function for the C-terminus, while amino acid residues His-19, Arg-46 and Asp-109 can be assigned to the active site in accordance with the structure.

## Results and discussion

In this work we have studied the function of the *E. coli *NAMNAT enzyme. Understanding the enzyme is necessary not only for its central role in metabolism but also for its possible role as a target for the development of new antibiotics. In an earlier study we initiated functional studies of two *E. coli *NAMNAT mutants [15]. One of the mutants, *nadD72*, is a frameshift mutation that changes the ten last amino acid residues of the protein and adds seventeen amino acids to the C-terminus [15]. When the amino acid sequences of bacterial NAMNATs are analyzed, two conserved residues, Tyr-205 and Ile-206 were found in the C-terminus. They are both located in the F helix of the *E. coli *enzyme, using the nomenclature given by Zhang *et al*. [10]. These two residues are changed in the NadD72 mutant. This lead us to ask whether it is the elongated C-terminus and/or the changed amino acids that cause the phenotype associated with the mutation. The other mutation, *nadD74*, changes Asp-13 to Val. The residue is next to the ATP-binding motif and is highly conserved [13].

To extend the study of NAMNAT we made three additional mutant alleles in the C-terminus and seven around the active site. For the *in vivo *studies we made a strain with a chromosomal deletion of the *nadD *gene, which makes it possible to study cloned, mutant enzymes *in vivo*. The gene on the chromosome was replaced by a cassette encoding chloramphenicol acetyltransferase [16] and the strain was called MS10. The gene *nadD *has been shown essential in *Salmonella typhimurium *[17]. However, some conflicting data on this matter was recently published [8]. The authors have isolated strains with transposon insertions affecting the *nadD *gene and the strains are viable on LB. The exact location of the insertion was not shown, moreover, attempts to delete the *nadD *gene from the chromosome failed. Our results are clear; we could only delete the *nadD *gene when the wild type *nadD *gene was present on a plasmid in the cell, thus, confirming the essentiality of the gene.

To study the mutant enzymes *in vitro *the different alleles were cloned in a vector under the control of the arabinose promoter. We first tried to fuse a His_6_-tag to the N-terminus of the proteins, but the expression level of the proteins was very low and some mutant proteins were undetectable when analyzed by Western blot (not shown). We changed to an IgG binding ZZ'-domain as a tag and the expression level increased considerably. A thrombin recognition sequence was engineered in the linker between the ZZ'-tag and the enzyme. However, removal of the ZZ'-tag from the purified proteins was not possible. Therefore, all investigated mutants and the wild type enzyme were assayed with the N-terminal ZZ-tag.

All enzymes were expressed as soluble proteins in strain TOP10. The final yield was typically 5–20 mg protein/l culture, similar to an earlier report [9]. The purity was about 90–95% as estimated by SDS-PAGE after Coomasie staining. The purified NadD72short protein gave two bands on the gel. One band had the correct enzyme size, the other, smaller, is probably a degradation product.

### Investigation of the C-terminus

Based on the *nadD72 *mutation, we designed the mutant NadD72short with the same change of the ten C-terminal amino acid residues as *nadD72*, while the length of the protein is the same as in the wild type enzyme. We also constructed two mutants where either Tyr-205 or Ile-206 is changed to alanine.

We have earlier shown that the intracellular level of NAD^+ ^correlates to growth ability on different media and at different temperatures [15]. Thus, we decided to investigate the effect on growth by the different mutant enzymes. Plasmids with either of the four mutant alleles, pZZNadD72, pZZNadD72short, pZZNadDY205A, pZZNadDI206A, or the wild type gene (pZZNadD) were transformed into strain MS10 with plasmid pKanNadD selecting for Ap^R^. Transformants were restreaked on LB with ampicillin and arabinose to induce expression of the respective *nadD *alleles. The transformants were tested for loss of Km^R ^to ensure plasmid exchange. Thereafter, MS10 with each respective plasmid were streaked on LB plates with or without arabinose and incubated at 30°C. The diameter of the colonies was measured. The results are shown in Table 4. Addition of 0.1 mM arabinose resulted in growth of all strains. In the absence of arabinose leakage expression of the wild type enzyme is enough to support normal growth while neither of the two mutant proteins NadD72 or NadD72short are active enough to do so. This indicates that it is the change in the last 10 amino acids and not the elongated C-terminus that impairs the enzyme. The two mutants with either of the two conserved residues changed, supported growth like the wild type enzyme. This shows that to affect enzyme activity more than one amino acid has to be changed in the C-terminus.

The same test was performed on minimal medium and it was found that NadD72 and NadD72short could not support growth in the presence of 0.1 mM arabinose. However, at 0.2 mM arabinose enough enzyme was produced to allow growth (not shown).

A test was also performed on strain MS10 containing plasmids carrying the *nadD *alleles without a ZZ'-tag. We could not detect any difference in growth behavior whether the *nadD *alleles were tagged or not (not shown). This makes us confident to use the ZZ'-tagged enzymes *in vitro*.

**Figure 2:**
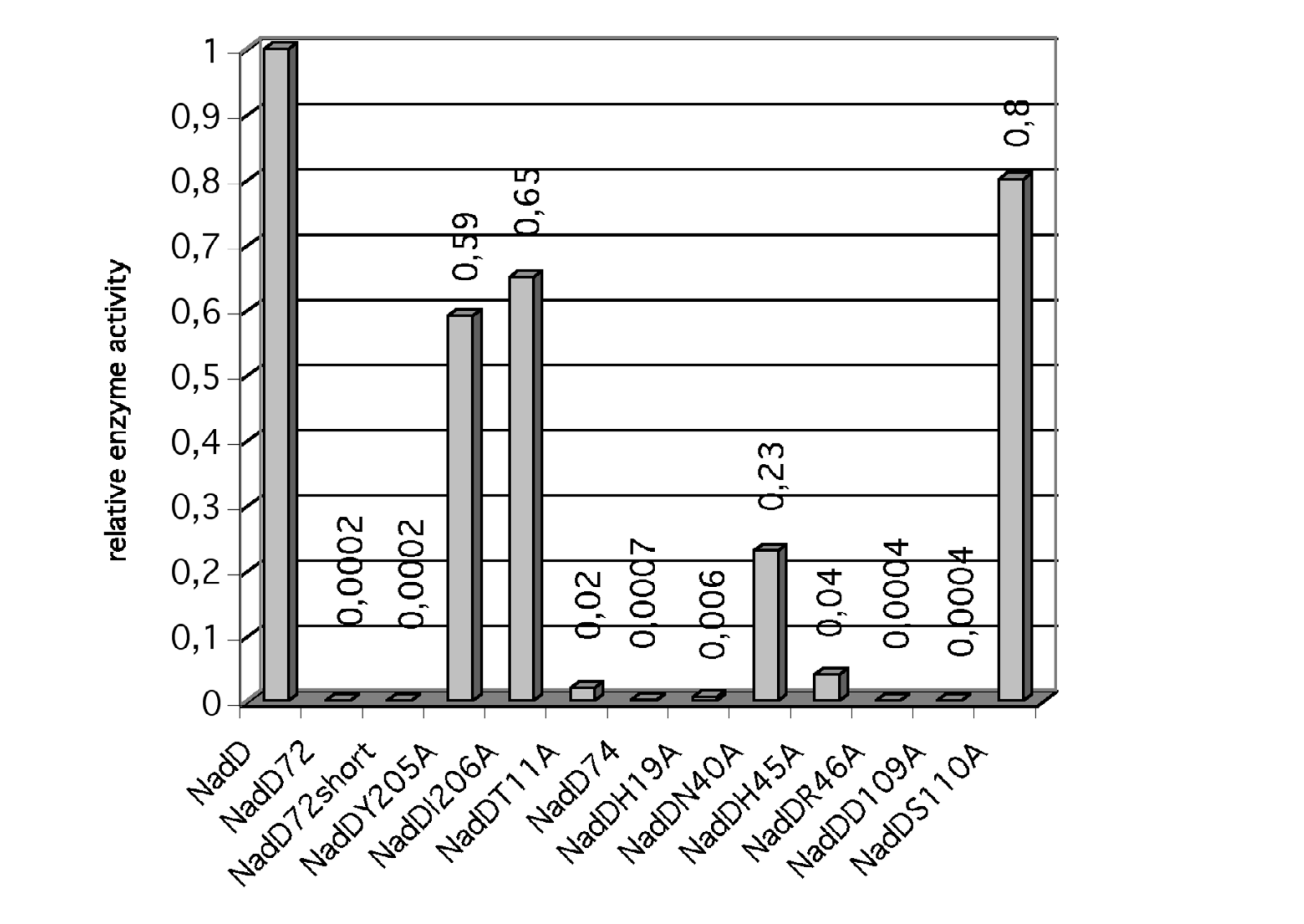
Relative enzyme activity of different NAMNAT mutants based on *in vitro *measurements. The results are based on at least three independent measurements with a standard error of the mean of less than 5%.

The enzyme activity of the mutants changed in the C-terminus were measured *in vitro*. The result can be seen in Figure 2. The activity of the wild type enzyme was set to 1. The enzymes NadD72 and NadD72short have almost no activity in good agreement with the *in vivo *phenotype. The other two mutants, NadDY205A and NadDI206A are less efficient than the wild type enzyme but not enough to show as a change in the growth phenotype.

The results obtained both *in vivo *and *in vitro *for NadD72short could indicate that it is not the extension of NadD72 that causes the deficiency in the protein but rather the change in the C-terminus. However, this conclusion is complicated by the finding during purification of NadD72short that two bands were visible on the protein gel, indicating instability of the protein. Thus, the results obtained for NadD72short are inconclusive.

Another possibility should also be considered. Since the *nadD72 *allele causes temperature sensitivity it is possible that the enzyme activity is lowered at 37°C the temperature at which the *in vitro *experiments were performed. To test this, we grew all mutants at 30°C, 37°C, and 42°C, respectively. We found that the Ts phenotype not only disappears in the presence of arabinose but that the cells grow better at the higher temperatures than at 30°C. Therefore, we do not think that the changes in enzyme activity are caused by changes in reaction temperature optimum.

To understand the role of the elongated C-terminus of the NadD72 mutant, we consider the role of the corresponding region of the human NMNAT. Human NMNAT has a 24 amino acid residues longer C-terminus than *E. coli *NAMNAT. It has been suggested that the C-terminus in human NMNAT plays a role in substrate recognition [18,19]. The NadD72 enzyme has 17 extra amino acid residues and it is possible that the extension interferes with substrate binding, which would lead to low enzyme activity. All we can say with certainty is that the C-terminus is important for stability of the protein and that the two conserved amino acid residues do not have a great influence on activity.

**Table 4:** 

MS10 + different plasmids	24 hours	48 hours
pZZNadD	1.1^†^	2.5
pZZNadD72	n.g.*	n.g.
pZZNadD74	n.g.	n.g.
pZZNadDY205A	1	2.2
pZZNadDI206A	1	2.3
pZZNadDT11A	0.2–0.5	1–1.5
pZZNadD74	0.6	1.2
pZZNadDH19A	0.2	0.6
pZZNadDN40A	1	2.1
pZZNadDH45A	0.8	1.4
pZZNadDR46A	0.2	1
pZZNadDD109A	0.1	0.3
pZZNadDS110A	1	2.2

^†^Colony size (diameter in mm); grown on LB without arabinose at 30°C; *n.g – no growth

### Investigation of the active site

**Figure 3:**
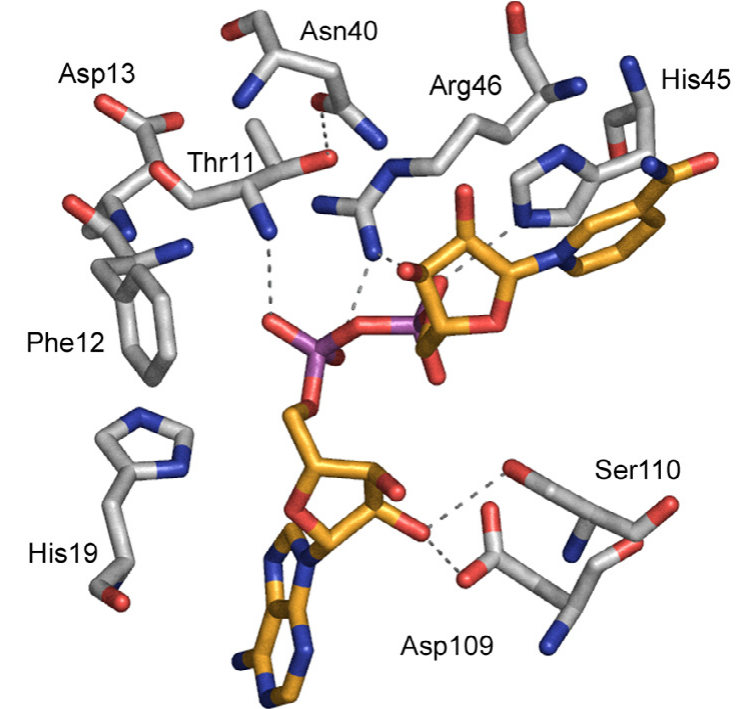
The active site of *E. coli *nicotinic acid mononucleotide adenylyltransferase with bound NAAD (yellow carbons). Amino acid residues (grey carbons) within 6 Å to NAAD are shown (tested in this study). Suggested H-bonds are marked by dotted lines [27]

The *nadD74 *mutation leads to an amino acid change in position 13 (Asp to Val). The mutated residue is two amino acids away from the ATP-binding motif, T/HXGH (position 16 to 19). Crystal structure information was used to decide which amino acid positions to mutagenize to learn more about the active site. Residues within 6 Å distance from the oxygens of the two adjacent phosphate-groups of the bound NAAD molecule are shown in Figure 3. Based on their close contact and H-bonding abilities Thr-11, His-19, Asn-40, His-45, Arg-46, Asp-109 and Ser-110 were selected for mutagenesis. All these residues were changed to alanine by site-directed mutagenesis. The recombinant proteins were cloned and analyzed, as were the C-terminal mutants.

First, growth on LB plates with and without arabinose was tested. The results are shown in Table 4. All strains grew in the presence of arabinose as expected. In the absence of arabinose MS10/pZZNadDN40A and MS10/pZZNadDS110A behaved basically as MS10 with the wild type enzyme while the other mutations affected growth on LB to a varying degree. Second, the enzymatic activity for the active site mutants was determined *in vitro*. The results are summarized in Figure 2. As with the C-terminal mutants, the correlation between the two experiments is very good.

Asp-109 and Ser-110 is located in the region connecting strand d and helix D of NAMNAT [10]. This region is one of three regions observed to undergo large conformational changes upon substrate binding. Interestingly, mutations of these two amino acids affect enzymatic activity very differently. On the one hand, binding of the substrate brings Ser-110 closer to the substrate. It is possible that the side chain of Ser-110 is H-bonded to the 2'-OH of AMP-ribose. However, mutation of Ser-110 to alanine resulted in an enzyme with 80% activity as compared to that of the wild type NadD. Our results indicate that the interaction between Ser-110 and the ribose is dispensable for substrate coordination. On the other hand, the change of the highly conserved Asp-109 had a severe effect on activity. Asp-109 has been proposed to form an H-bond to the 2'-OH group of AMP [10]. It was also suggested by the same authors that the carboxylate oxygen of the residue might be involved in the coordination of a Mg^2+ ^ion shown to be important for the enzyme function [10,12]. The location of Asp-109 is ideal to position an Mg^2+ ^ion which could act as a Lewis acid stabilizing the transition state in the transesterification reaction [10]. However, the functional importance of this conserved residue has not been investigated earlier. The serious decrease in enzymatic activity (0.038%) that we observe supports the above mechanism and serves as an experimental evidence for the involvement of Asp-109 in the catalysis.

The Thr-11 main chain nitrogen is H-bonded to the AMP phosphate in the crystal structure of the NadD-NAAD complex. This H-bond is expected to be independent of the side-chain character of the amino acid. However, the NadDT11A mutant leads to decreased enzyme activity (2%, Figure 2). The explanation might be that the hydroxyl group of Thr-11 forms an H-bridge to Asn-40, which has been shown to interact with 2'-hydroxyl group of NAMN-ribose [10]. Such an interaction could contribute to substrate binding and coordination. The change of Asn-40 to alanine leads to a decreased enzyme activity (23%, Figure 2). We conclude that the disruption of this H-bonding network might lead to inefficient substrate coordination. Therefore, the effect on enzyme activity by the T11A and/or N40A mutation is possibly indirect.

The mutation D13V (NadD74) was found to lead to decreased activity of the enzyme. A similar mutation has been described in CTP:glycerol-3-phosphate cytidyltranferase that is a member of the same enzyme super family [20]. The residue Asp-11 (which corresponds to Asp-13 in NAMNAT) was changed to alanine and enzyme activity was severely reduced. Both valine and alanine are hydrophobic amino acids and it is possible that the change disturbs ATP binding; resulting in lower enzyme activity. It is not clear whether the role of Asp in this position is catalytical or structural.

The role of the second histidine (His-19) in the conserved T/HXGH motif has been tested in several studies and been shown to play a role in ATP-binding and stabilization, but the role for the two histidine residues can vary between enzymes [14,21]. Our results confirm the previous observations. The mutation H19A leads to a decrease in enzyme activity to 0.62% of the wild type activity.

Amino acid residues His-45 and Arg-46 are part of a flexible loop in the NadD enzyme, which moves upon substrate binding. The two residues are conserved within NMNAT from Bacteria and Eukarya but not in the known archaeal enzymes. His-45 is involved in a hydrophobic stacking interaction with the pyridine ring in NAMN and is also likely to form an H-bond with the NAMN phosphate group. Histidines are often involved in acid base catalysis, and prone to activate nucleophiles by abstracting a proton. The H45A change led to an enzyme with 4% residual activity, while the R46A mutant was the most affected of all tested active site mutants. If the role of Arg-46 in the substrate binding loop was simply to protect the bound substrates, higher rates of ATP hydrolysis is expected in the case of the R46A mutant. Since the R46A mutant did not produce AMP as by-product in the *in vitro *experiment, the role of this arginine side chain must be more than simply protecting the bound substrates from water molecules. The guanidinium group of Arg-46 lies in an ideal position to serve as a positively charged moiety that stabilizes the juxtaposed and negatively charged phosphates of the substrate molecules, as well as of the product NAAD. The archaeal orthologues lack the precise sequence homology in the sequence aligned to the H45-R46, but they have a conserved arginine (Arg-8 in *Methanococcus jannaschii*) that occupies the same position with its side chain in the ATP-enzyme complex as that of Arg-46 in the *E. coli *NadD enzyme [10,12]. On the other hand another hypothesis should be considered as well. Mutational studies on NMNAT from *Methanobacterium thermoautotrophicum *indicate that the archaeal enzyme is involved in the reaction merely by placing the substrates in an ideal position [14]. Combining the facts above, we conclude that Arg-46 in *E. coli *NadD plays an important role in stabilizing the two adjacent, negatively charged, phosphate moieties during catalysis. A similar role might be attributed to Arg-8 in the archaeal counterparts but this has to be tested.

## Conclusion

We have investigated mutants in both the C-terminus and the active site of *E. coli *NAMNAT in two assay systems; effect on growth on plates and *in vitro *activity of the enzyme. Correlation between the two data sets is very good and shows that there is a distinct threshold where there is enough activity to support growth.

The data obtained are not enough to assess the function of the C-terminus; more work is needed. As for to the active site we have found that amino acid residues His-19, His-45, Arg-46 and Asp-109 are likely needed for catalysis, while Asp-13 probably affects substrate binding indirectly. We have also shown the essentiality of the *nadD *gene in *E. coli*.

## Methods

### Bacterial strains and media

Bacterial strains and plasmids used in this work are listed in Tables 1 and 2, respectively. Luria-Bertani (LB) medium and M9 minimal medium were prepared according to Miller [22]. The concentrations of antibiotics were, 15 μg/ml chloramphenicol, 200 μg/ml ampicillin (Ap) and 50 μg/ml kanamycin (Km).

**Table 1:** Strains used in this study

Strain	Genotype	Source
MRA530	*rph nadA*::Tn*10 gal490*(?) λ cI^857 ^Δ(*cro*-*biol*)(?)	MRA strain collection
RI8	*ara *Δ(*gpt-lac*)_5 _*nadD72 zbe280*::Tn*10*	[15]
RI10	*ara *Δ(*gpt*-*lac*)_5 _*zbe280*::Tn*10*	[15]
RI12	*ara *Δ(*gpt*-*lac*)_5 _*nadD74 zbe280*::Tn*10*	[15]
MS10	as RI10 but *nadD*::Cm	This study
TOP10	F^- ^*mcr*A Δ(*mrr*-*hsd*RMS-*mcr*BC) φ80*lacZΔM15 *Δ*lacX74 deoR recA1 araD139 *Δ(*ara*-*leu*)7697 *galU galK rpsL endA1 nupG*	Invitrogen, Carslbad, CA
DH5α	*F*^-^,φ80d*lacZΔM15*, Δ(*lacZYA*-*argF*)U169, *deoR*, *recA1*, *endA*, *hsdR17*(rk^-^, rk^+^), *supE44*, *gyrA96*, *relA1*	[25]

**Table 2:** Plasmids used in this study.

Plasmid name	description
pEZZ18	Pharmacia Biotech/GE Healthcare
pUC4K	Pharmacia Biotech/GE Healthcare
pBADmyc-hisA	Invitrogen (Ap^R^)

pBAD-Kan	As pBADmyc-hisA but Km^R^
pKanNadD	wild type *nadD *in pBAD-Kan

pNadD	wild type *nadD*
pNadD72	*nadD72*(D13V)
pNadD74	*nadD74*
pNadD72short	*nadD72 *without the extension
pNadDT11A	*nadD *with mutation T11A
pNadDH19A	*nadD *with mutation H19A
pNadDN40A	*nadD *with mutation N40A
pNadDH45A	*nadD *with mutation H45A
pNadDR46A	*nadD *with mutation R46A
pNadDD109A	*nadD *with mutation D109A
pNadDS110A	*nadD *with mutation S110A
pNadDY205A	*nadD *with mutation Y205A
pNadDI206A	*nadD *with mutation I206A
pZZNadD	pNadD + ZZ'-tag N-terminal fusion
pZZNadD72	pNadD72 + ZZ'-tag N-terminal fusion
pZZNadD74	pNadD74 + ZZ'-tag N-terminal fusion
pZZNadD72short	pNadD72short + ZZ'-tag N-terminal fusion
pZZNadDT11A	pNadDT11A + ZZ'-tag N-terminal fusion
pZZNadDH19A	pNadDH19A + ZZ'-tag N-terminal fusion
pZZNadDN40A	pNadDN40A + ZZ'-tag N-terminal fusion
pZZNadDH45A	pNadDH45A + ZZ'-tag N-terminal fusion
pZZNadDR46A	pNadDR46A + ZZ'-tag N-terminal fusion
pZZNadDD109A	pNadDD109A + ZZ'-tag N-terminal fusion
pZZNadDS110A	pNadDS110A + ZZ'-tag N-terminal fusion
pZZNadDY205A	pNadDY205A+ ZZ'-tag N-terminal fusion
pZZNadDI206A	pNadDI206A + ZZ'-tag N-terminal fusion

All plasmids below line three were made in this study. All plasmids below line five are derivatives of pBADMyc-hisA.

Standard recombinant DNA techniques were used for cloning of DNA [23]. *E. coli *strain DH5α was used as a recipient for cloned DNA. Restriction and modification enzymes were purchased from New England Biolabs, Amersham Pharmacia Biotech or Life Technologies. DNA fragments were separated by agarose gel electrophoresis, excised and purified using the Qiaex II Gel Extraction Kit (Qiagen). Oligonucleotides were purchased from MWG Biotech. Plasmid DNA was purified with QiaPrep Kit (Qiagen). MWG Biotech did DNA sequencing. ATP and NAMN were purchased from Sigma.

### Construction of a *nadD* strain and a plasmid-exchange system

Primers used in this study are listed in Table 3. Before deleting the *nadD *gene from the chromosome we had to clone the wild type gene. The *nadD *wild type gene was amplified from RI10 with primers PARA1 and PARA2 and cloned into plasmid pBAD-Kan. The plasmid was named pKanNadD. The plasmid pBAD-Kan is identical to pBADmyc-hisA but with a kanamycin resistance gene from pUC4K inserted into the ampicillin resistance gene. Thus, pBAD-Kan confers kanamycin resistance and not ampicillin resistance.

**Table 3:** Oligonucleotides used in this study.

Primer	sequence
PNadko1b	5'- ATAAACCCCTGGCGGACGTATTTATCGACGGTTGATCATATGAATATCCTCCTTAG-3'
PNadko2b	5'- TGGTCGCCGAGATGTTAAACCACGGCGTTTCAGCCAGTGTAGGCTGGAGCTGCTTC-3'
PARA1	5'-AACCATGGAATCTTTACAGGCTCTGTTTGGC-3'
PARA2	5'-TTAAGGTACCGTAACGACAGGTATCAGCGAT-3'
PD1	5'-TTAAGGTACCTCACCATGACGAATTAACCAC-3'
PD72short	5'-TTAAGGTACCTCAAGCGATACAAGCCTTGTT-3'
T11A*	5'-ACAGGCTCTGTTTGGCGGC**GCC**TTTGATCCGGT-3'
H19A*	5'-GATCCGGTGCACTATGGT**GCT**CTAAAACCCGTGGAA-3'
N40A*	5'-CGGGTCACAATCATCCCT**GCT**AATGTTCCTCCGCAT-3'
H45A*	5'-CTAATAATGTTCCTCCG**GCT**CGTCCCCAAGCCGGAAGC-3'
R46A*	5'-CTAATAATGTTCCTCCGCAT**GCT**CCCCAGCCGGAAGC-3'
D109A*	5'-TTTATTATTGGTCAG**GCT**TCACTGCTGACCTTTCCG-3'
S110A*	5'-ATTATTGGTCAGGAT**GCA**CTGCTGACCTTTCCGACC-3'
Y205A*	5'-GGAACCGGTACTGACT**GCC**ATTAACCAACAAGGCTTG-3'
I206A*	5'-GGAACCGGTACTGACTTAC**GCT**AACCAACAAGGCTTG-3'

* oligonucleotides used for site directed mutagenesis. The mutated codons are shown in bold, the changed nucleotides are underlined.

To delete the *nadD *gene from the chromosome we used linear DNA transformation and the λ red recombination system [16]. Primers used for amplification of the chloramphenicol acetyltransferase gene with homologies to the ends of *nadD *were PNadko1b and PNadko2b. The amplified linear DNA was electroporated into strain MRA530/pKanNadD and chloramphenicol resistant (Cm^R^) colonies were selected. Recombinants were checked for proper exchange by PCR amplification and sequence verification. A P1 phage lysate made on one such recombinant was transduced to RI10/pKanNadD selecting for Cm^R^. Transductants were verified by PCR, one clone was kept and named MS10. The plasmid could now be exchanged for a pBADmyc-hisA (Ap^R^) plasmid carrying different *nadD *alleles. The exchange relies on the incompatibility of pKanNadD and pBADmyc-hisA.

### Construction of expression vectors with different *nadD *alleles

The *nadD72 *allele from RI8 was amplified with primers PARA1 and PD1, the *nadD74 *allele from RI12 with primers PARA1 and PARA2 and the *nadD72short *allele from RI8 with primers PARA1 and PD72short. The *nadD72short *allele has the same change at the C-terminus as the *nadD72 *allele but with the same length as the wild type allele. Site-directed mutagenesis was performed on the cloned wild type allele using QuikChange Site-Directed Mutagenesis Kit (Stratagene, La Jolla, CA). Oligonucleotides used are listed in Table 3.

All allele variants were cloned in the expression vector pBADmyc-hisA under the control of an arabinose-promoter using the *Nco*I and *Kpn*I sites. The resulting plasmids are listed in Table 2. Thereafter all alleles were fused at their 5'-end to a fragment containing an IgG binding ZZ'-domain from the pEZZ18 vector. This set of plasmids has the same origin of replication as the pKanNadD but they confer ampicillin resistance (Table 2). This makes it possible to exchange plasmid pKanNadD in the MS10 strain for plasmids carrying mutant *nadD* alleles.

### Protein expression and purification

*E. coli *TOP10 cells containing plasmids with respective *nadD *allele (pZZNadDXX) were grown in LB medium at 30°C. When the cultures reached OD_550 _0.5, arabinose was added to a final concentration of 0.4 mM. After ~4 h growth, the cultures were quickly chilled on ice and harvested by centrifugation. Pellets were stored at -20°C. The frozen pellets were thawed, resuspended in 10 × TST (0.5 M Tris pH 7.4, 1.5 M NaCl, 5% w/v Tween 20), lysozyme (1 mg/ml), DNase I (20 μg/ml) and RNase A (20 μg/ml). After incubation at 37°C and several freezing (in liquid nitrogen) and thawing cycles, samples were sonicated and the lysate was cleared by centrifugation. Proteins were purified as described [24]. The solvent was changed to reaction buffer [9] on a NAP5 desalting column (Amersham Biosciences, Uppsala, Sweden). Purification efficiency was monitored by SDS-polyacrylamide gel elecrophoresis (PAGE) and stained by Coomasie blue. The protein concentration was determined by UV spectrophotometry at 280 nm [26].

### Assay of enzymatic activity

*In vitro *enzymatic activity of NAMNAT was determined as described [9]. The substrate concentrations were 2 mM ATP and 1 mM NAMN and the reaction was carried out at 37°C. The enzyme concentration was 1 μg/100 μl. Reactions were terminated by immersing the tubes in boiling water for 5 minutes. They were thereafter cooled on ice and filtered through Nanosep 10 K microcentrifugal devices to take away the enzyme (Pall, Ann Arbor, MI). 20 μl aliquots were analyzed by high-pressure liquid chromatography on a Gilson LC system by using Supelcosil LC-18-T 15 cm by 4.6 mm column (Supelco, Bellefonte, PA).

The product formation was monitored at 254 nm as a function of time and the initial reaction rate was calculated from the slope of the curve. The rate for the wild type enzyme that was set to 1, rates for the mutant enzymes were correlated to this.

## Authors' contributions

RS expressed and prepared all the mutant enzymes, discussed the work and helped to draft the manuscript. MS did all the rest of the lab work, designed the experiments and drafted the manuscript. MRA conceived of the study, participated in its design and coordination, and drafted the manuscript. All authors read and approved the final manuscript.
